# COVID-19 and Oral Lichen Planus: Between an “Intriguing Plot” and the “Fata Morgana Effect”

**DOI:** 10.3390/jcm12144829

**Published:** 2023-07-21

**Authors:** Gaetano Scotto, Vincenzina Fazio, Salvatore Massa, Lorenzo Lo Muzio, Francesca Spirito

**Affiliations:** 1 Infectious Diseases Unit, University Hospital “OORR” Foggia, 71122 Foggia, Italy; gaetano.scotto@unifg.it; 2 Clinical Chemistry Laboratory, Virology Unit, University Hospital “OORR” Foggia, 71122 Foggia, Italy; vincenzina.fazio@unifg.it; 3 Department of Agriculture, Food, Natural Resource and Engineering, University of Foggia, 71122 Foggia, Italy; salvatore.massa@unifg.it; 4 Department of Clinical and Experimental Medicine, University of Foggia, 71122 Foggia, Italy; lorenzo.lomuzio@unifg.it

**Keywords:** COVID-19, SARS-CoV-2, oral lichen planus

## Abstract

The COVID-19 pandemic, caused by the SARS-CoV-2 virus, has led to significant morbidity and mortality worldwide since its declaration as a global pandemic in March 2020. Alongside the typical respiratory symptoms, unusual clinical manifestations such as oral lichen planus (OLP) have been observed. OLP is a chronic inflammatory mucocutaneous dermatosis that results from a cell-mediated reaction, and its pathogenesis involves the loss of immunological tolerance. OLP has been associated with several triggering factors, such as certain drugs, stress, smoking, and even some viruses. Exposure to the spike protein antigen of SARS-CoV-2 during an infection can trigger autoimmune reactions and lead to the onset or flare of OLP. The E3 protein ligase TRIM21, which is identified in the lamina propria of OLP lesions, is overexpressed in COVID-19 patients and plays a critical role in autoimmune pathologies. Furthermore, the psychological stress of the lockdown and quarantine can be a trigger for the onset or exacerbation of OLP. However, the diagnosis of OLP is complex and requires a biopsy in order to confirm a clinical diagnosis, rule out other pathologies, and establish the most appropriate therapeutic procedure. Further research is needed to understand the potential link between Co-19 and OLP.

It has been about three years since the World Health Organization (WHO) declared coronavirus disease-19, COVID-19 (Co-19), as a global pandemic on 11 March 2020. Co-19 is an acute respiratory disease that is caused by the SARS-CoV-2 virus. COVID-19 exhibits a wide range of clinical manifestations that vary from asymptomatic or mildly symptomatic cases, which feature upper respiratory tract involvement, to severe forms that feature lung impairment and life-threatening conditions that are caused by critical respiratory distress. In mild cases, Co-19 symptoms are similar to the flu, and the most commonly observed symptoms of COVID-19 include fever, fatigue, dry cough, headache, hemoptysis, anorexia, and sore throat [1]; in the most critical cases, it is a systemic disease with severe acute respiratory syndrome. However, in addition to the “classic” clinical features, there have been reports linking Co-19 infection or Co-19 therapy to less common pathologies, including atypical manifestations that affect various systems such as the neurological, psychiatric, neuropsychiatric, cardiovascular, gastrointestinal, and dermatological systems [2]. The virus enters host cells through the binding of the homotrimeric spike glycoprotein on the viral surface to the angiotensin-converting enzyme 2 (ACE2) receptor, which is found in different organs and systems of the human body [3]. This mechanism of viral entry contributes to the characteristic multiorgan tropism of the virus. SARS-CoV-2 demonstrates a particular tropism for head and neck tissues, and it leads to distinct extrapulmonary manifestations that have become notable features of the disease [4]. These manifestations include dysfunctions in the sense of smell (anosmia) and taste (ageusia, dysgeusia) [5]. Indeed, the receptors and enzymes that are required for SARS-CoV-2 cell entry, such as the ACE2 receptor [6], transmembrane protease serine 2 (TMPRSS2), and furin [7,8], are highly expressed in oral and nasal cavity tissues, including the epithelial cells of the tongue, taste buds (particularly fungiform papillae), major and minor salivary glands, and nasal mucosa. The virus enters target cells through the interaction between the spike protein (S protein) and the ACE2 receptor [6,9], and TMPRSS2 plays a priming role in this process [5]. Therefore, this specific tropism manifests through the development of oral cavity pathologies [10,11,12] and other maxillofacial conditions, such as the well-known association with mucormycosis [13,14]. Indeed, during SARS-CoV-2 infection, the oral mucosa can serve as a potential route for the virus, and regional pathologies in the oral cavity may arise [15,16,17]. These pathologies can manifest as ulcerative lesions, vesicles, blisters, petechiae, erythema multiforme-like lesions, aphthous-like lesions, and herpetiform lesions, which typically concurrently appear with general symptoms or within one week [10,17,18,19,20]. Oral manifestations can also be a result of COVID-19 therapies, such as the long-term use of antibiotics or mechanical ventilation devices in severely affected patients, which can lead to Candida infections in the form of red or white plaque lesions [21]. Delayed onset oral lesions, including ulcerations, bullous angina, petechiae, and late-onset erythema multiforme-like lesions, have also been reported following the initiation of therapy [20]. The prolonged hospitalization of patients can contribute to poor oral hygiene and be combined with compromised overall health, states of immunosuppression, and physical and psychological stress, which can lead to the development of plaque-related diseases, such as ulcer-necrotic gingivitis [22]. Regarding certain oral cavity diseases, some reports have recently appeared in the literature that must be examined with a touch of curiosity and pinch of skepticism as they discuss the onset or exacerbation of oral lichen planus (OLP) during Co-19 infection [23].

OLP—a clinical variant of lichen planus (LP), which is a chronic inflammatory mucocutaneous dermatosis of immune etiology—results in an inflammatory state that affects the stratified squamous epithelium of the oral cavity and the underlying lamina propria [24]. OLP is a cell-mediated reaction in which CD8 cytotoxic lymphocytes play the primary role against an unknown keratinocyte antigen that is no longer recognized as self. Consequently, the lesions can be seen as the manifestation of various oral diseases and conditions that are caused by the loss of immunological tolerance. Both LP and OLP can arise or be exacerbated in the presence of triggering factors such as certain drugs (non-steroidal anti-inflammatory drugs (NSAIDs), antihypertensives, antimalarials, etc.), psycho-organic stress, smoking, and even some viruses [25,26,27]. In fact, an association between the presence of OLP and infection by citomegalovirus (CMV), herpes simplex (HSV) type 1, 4, and 6, hepatitis B virus (HBV), papilloma virus and, above all, hepatitis C virus (HCV) has been identified [26,27]. This is probably due to the extra-hepatic manifestations of HCV, such as cryoglobulinemia [27]. Data on the inductive mechanisms of the potential triggers and on the identities of the target antigens are still not conclusive; however, the immune dysregulation alludes to the hypothesis of an autoimmune-based chronic inflammatory reaction.

As far as Co-19/OLP is concerned, this hypothesis seems plausible, as immune dysregulation with hints of autoimmunity would seem to be a common finding by it playing a fundamental role in the pathogenesis of OLP and underlying the cytokine storm that is a hallmark of Co-19. There are several theories linking the pathogenesis of oral lichen planus with Co-19, starting from the fact that SARS-CoV-2 infection has been linked to an imbalance in the signaling pathway of the mammalian target of rapamycin (mTOR), which is known to contribute to the abnormal proliferation of T-cells and the development of OLP. It has been hypothesized that exposure to the spike protein antigen during a Co-19 infection can trigger an autoimmune reaction. Increased levels of inflammatory mediators such as cytokines, matrix metalloproteinases, and chemokines, as well as the recruitment and alteration of the activity of cytotoxic CD8+ T cells (CTLs), would mediate the onset or flare of OLP [28]. Furthermore, Co-19 seems to trigger the overexpression of the tripartite motif-containing protein 21 (E3 protein ligase TRIM21), which belongs to a tripartite motif protein family that is encoded by gene 21 and is an important autoantigen in autoimmune pathologies (Sjogren syndrome, systemic lupus erythematosus (SLE)). This stimulates CTL and increases cytokine production with enhanced IL-6 secretion; the trait d’union would be the identification of E3TRIM21 as also being in the lamina propria of OLP lesions [29,30]. Furthermore, the cytokine storms that are characterized by increased levels of interleukin-1 (IL-1), interleukin-6 (IL-6), interleukin-12 (IL-12), interferon y (IFN-γ), and tumor necrosis factor α (TNF-α) are a prominent feature of SARS-CoV-2 infection, and they contribute to tissue damage and inflammation; it should also be added that, in COVID-19 patients, there is a high level of interleukin-2 (IL-2), interleukin-4 (IL-4), interleukin-7 (IL-7), interleukin-10 (IL-10), interleukin-13 (IL-13), and interleukin-17 (IL-17) [31]. Several interleukins (ILs), including IL-1, IL-2, IL-4, IL-5, IL-6, IL-8, IL-10, IL-12, IL-17, and IL-18, have been implicated in the development and progression of OLP [32]. Genetic variations in IL-18, TNFα, IFN-γ, IL-10, IL-17, IL-1ß, IL-12, IL-8, and IL-4 have also been associated with OLP [28]. These cytokines damage the basement membrane and cause extensive tissue destruction, which results in the formation of observable lesions. The increased levels of cytokines, malfunctioning T-cells, and activation of TRIM21 in individuals that are affected by or recovering from COVID-19 may make them more vulnerable to developing oral lichen planus.

A further hypothesis, although very suggestive, is that Co-19 may not directly contribute to OLP, but when combined with psychological stress with depressive elements that are linked to the lockdown and/or quarantine, it can be a trigger for the onset or exacerbation of OLP. It is known that stress can be a cause for the alteration of various parameters of the endocrine system and immune dysregulation in subjects that are affected by OLP [25,33]. In fact, a high increase in stress can activate the sympathetic nervous system and the hypothalamic–pituitary–adrenocortical axis, causing a significant influx of inflammatory cytokines such as interleukin-6 (IL-6), interleukin-1 (IL-1) and tumor necrosis factor (TNF), as well as an alteration of CTL activity. As a result of this influx, changes in kynurenine metabolites lead to neurotoxic changes in the brain. This is likely to result in an autoimmune reaction, which can trigger the onset or exacerbation of OLP [34,35]. This seems to happen preferentially in women, as demonstrated in an Indian study that was carried out on socially frail women during the pandemic. The reason for this is that female hormonal changes, in addition to the increase in cytokines, make them more susceptible to systemic disorders that are induced by a state of stress or depression [36,37]. Considering the above, there is a high probability of an “intriguing mix” between the two pathologies of Co-19 and OLP; however, in medicine, there is usually a catch.

The diagnosis of OLP is typically based on a combination of clinical objectivity and histopathological features. Exclusively defining the diagnosis on clinical criteria presents critical issues, as studies have shown possible interpretative variability. The lesions that present themselves in OLP in an erosive, atrophic, bullous, reticular, and plaque form can, in fact, also be found in other clinical conditions, such as lupus erythematosus, syphilis, candidiasis, aphthous ulcers, pemphigoid mucosa, and carcinoma of the oral cavity [24]. Therefore, a biopsy is necessary to confirm a clinical diagnosis to exclude other pathologies and establish the most appropriate therapeutic procedure. In 2003, van der Meij and van der Waal [38] proposed a modification of the diagnostic criteria that were previously recommended by the WHO in order to reduce this variability. The complete correspondence of the clinical criteria (presence of bilateral lesions, presence of reticular striae) and histopathological criteria (presence of a well-defined area of cellular infiltration with a “band” arrangement that is limited to the superficial part of the connective tissue; signs of degeneration in the layer of basal cells; absence of epithelial dysplasia) constituted and still constitutes the necessary check for a diagnosis of OLP [38,39]. After analyzing the scientific literature published between 2020 and 2022, it was possible to highlight the prevalence of the Co-19/OLP association and the criteria that led to the diagnoses.

We screened Medline via the PubMed, Web of Science, Scopus and Google scholar databases for articles investigating the association between COVID and oral lichen that were published between 1, January 2020 and 31 May 2023. Several combinations of keywords were used in the following orders to conduct the search strategy: (1) “Lichen Planus’’ OR “Oral Lichen Planus’’; and (2) “COVID-19” OR ‘’Co-19′’ OR “SARS-CoV-2” OR “Coronavirus”.

All clinical studies, case series, and case reports that reported cases of OLP in association with COVID-19 were considered as potentially admissible. To date, 17 cases of post-infection OLP have been described, including individual case reports, which constitute the vast majority, as well as case series and observational studies. Only five cases provide more detailed clinical characteristics, which are reported in Table 1 [40,41,42,43]. From a strictly clinical-morphological point of view, four out of five subjects presented a papulo-reticular form with evident Wicham’s striae and one out of five subjects presented painful erosive areas surrounded by white radiating striae. Three out of five patients were symptomatic, and three patients had cutaneous involvement. In one case, there was information about the histopathology included: a hyperparakeratinized, stratified epithelium; partial ulceration; mild degree of acanthosis; liquefactive degeneration of basal cells; and prominent band-like sub-epithelial lymphocytic infiltration. The remaining 12 cases were part of an observational study [23] that considered all oral lesions that were found in a cohort of patients with COVID-19. In this study by Fidan et al. [23], oral lichen planus represented 20.6% of the lesions found in the 74 included patients. Considering all the cases, the manifestations of significance were observed in various locations of the oral cavity, where it was observed in the tongue for four cases, buccal mucosa for nine cases, gums for five cases, and palate for one case [23,40,41,42,43,44].

The diagnosis seems to have been made by clinical observation in 16 cases and only in 1 case was it confirmed by a histopathology from oral biopsy; the vast majority of authors did not mention a biopsy of the lesions, so it is assumed that the diagnoses were only based on clinical observations. Describing it as OLP is questionable in such cases, as various oral pathologies can present similar clinical observations. Furthermore, in 12 out of 17 cases, the descriptions of the lesions were directed towards single localizations, an aspect that should not be typical of OLP. In fact, one of its most relevant clinical characteristics is its frequent bilateral expression and ability to spread to several areas in the oral cavity; this aspect often helps the clinician to arrive at a correct diagnosis of OLP and exclude, in differential diagnosis, other lesions with unique and monolateral topographical characteristics. From this literature analysis, it is evident that there is a lack of data along with reporting bias, as confirmed by a recent literature review that indicates that, when considering all the data regarding lichen planus occurring after infection or vaccination, there are more cases that have been reported post-vaccination than post-infection in the literature [45].

To conclude, while our intention is not to criticize the scientific rigor of our colleagues’ research, the critical issue with these reports lies in the small number of cases and the limited use of histological tests for confirmation. These biases, when combined with other clinical descriptors, could cause confusion and make the diagnosis extremely uncertain, a drawback of which that is also highlighted by some authors of these reports. Therefore, additional reports of cases with confirmatory histological tests are required. This will lead to a discussion of an “intriguing Co-19/OLP plot” and not of a “Fata Morgana mirage effect”, which causes us to see what we want to see and not what it actually is.

## Figures and Tables

**Table 1:** Case report and cases series reporting OLP after COVID-19 infection.

Article	Case	Sex	Age (Years)	After Infection, Days to Onset of Symptoms	Location of Lesions	Lesions Morphology and Symptomatology	Cutaneous Manifestation	Histopathology (Oral Biopsy)	Treatment for Oral Lesions	Outcomes of Oral Lesions
[40]	Case 1	F	56	42 days	Bilateral buccal mucosae	Papules with lace-like pattern	Widespread papulosquamous eruption on the trunk	NR	NR	NR
[41]	Case 2	F	52	NR	Bilateral buccal mucosae	Papules with lace-like pattern	Black annular plaque with a whitish rim on the right shin that measured 2 cm in diameter	NR	None	NR
[42]	Case 3	M	63	30 days	Dorsum linguae + buccal mucosae	Painful erosive areas surrounded by white radiating striae	Brown pruritic macules on the flexure surface of the arm	Hyper-parakeratinized stratified epithelium, partial ulceration, mild degree of acanthosis, liquefactive degeneration of basal cells, and prominent band-like sub- epithelial lymphocytic infiltration	Topical corticosteroids	Symptom remission and reduction of the extent of lesion (4 weeks)
[43]	Case 5	M	41	14 days	Buccal and gingival mucosae	Lichenoid striations with erythema + oral sensitivity	NR	NR	Topical corticosteroids	Symptom remission (4 weeks)
										Reduction of the extent of lesions
	Case 6	F	56	NR	Bilateral buccal mucosa	Lichenoid striations with erythema + oral sensitivity	NR	NR	Topical corticosteroids	NR

## Data Availability

No new data were created.
